# Bio-oil modified binder derived from cotton stalks as an eco-friendly alternative binder for flexible pavements

**DOI:** 10.1038/s41598-024-62652-5

**Published:** 2024-06-12

**Authors:** Sahib Ullah, Syed Bilal Ahmed Zaidi, Diyar Khan, Ayyaz Fareed, Mohd Rosli Mohd Hasan, Abdalrhman Milad, Basit Ali

**Affiliations:** 1 Department of Civil Engineering, University of Engineering and Technology, Taxila, Pakistan; 2 Doctoral School, Silesian University of Technology, Akademicka 2a, 44-100 Gliwice, Poland; 3 Sustainable Asphalt Research Group (SARG), School of Civil Engineering, Universiti Sains Malaysia (Engineering Campus), 14300 Nibong Tebal, Penang Malaysia; 4 Department of Civil and Environmental Engineering, College of Engineering and Architecture, University of Nizwa, 616 Birkat-al-Mouz, Nizwa, Oman; 5 School of Highway Engineering, Chang’an University, Xi’an, PR China

**Keywords:** Bio-oil modified binder, Cotton stalk, Environmental sustainability, Alternative materials, Engineering, Civil engineering

## Abstract

Scientists and engineers encounter considerable environmental and economic obstacles stemming from the depletion of crude oil or petroleum fossil fuel reservoirs. To mitigate this challenge, alternative solutions like bio-oil-modified binder derived from biomass have been innovated. This research aims to examine the feasibility of using bio-oil-modified binder obtained from cotton stalk waste as a modifier. Various mechanical and physical tests, including penetration, softening point, ductility, and dynamic shear rheometer tests, were conducted on asphalt binder incorporating 5% and 10% bio-oil-modified binder. Wheel tracker, four-point beam fatigue, and dynamic modulus tests were used to evaluate asphalt mixture performance, including rutting, fatigue, and dynamic stiffness. A rolling bottle test (RBT) and asphalt binder bond strength (BBS) were used to assess moisture susceptibility. A bio-oil-modified binder enhanced ductility and penetration characteristics while reducing the softening point. With the addition of a bio-oil-modified binder, stiffness was reduced in parameters such as complex shear modulus and phase angle. In fact, for both specimens containing 5% and 10% bio-oil-modified binder, statistically significant differences were observed among the measured samples. As a result of this reduced stiffness, the modified asphalt binder is more suitable for low-temperature applications. Additionally, 5.8% increased at 10% and 3.1% at 5% CS. Bio-oil-modified binder, compared to virgin mixtures, supports equal rut resistance. However, the RBT and BBS tests revealed that the addition of bio-oil-modified binder increased the susceptibility of conventional asphalt binder to moisture. The findings suggest that bio-oil-modified binder can enhance asphalt binder properties in low-temperature regions, but further research is needed to improve moisture resistance.

## Introduction

On a global scale, cotton stalk (CS) is an important agricultural crop. Cotton is grown on more than 12 million hectares of land in 80 countries across the world^1^. China produces 40 million metric tons of cotton stalk annually, making it the largest cotton producer in the world^2^. Agricultural waste, typically disposed of in landfills, poses environmental contamination risks, particularly during the rainy season when it can mix with groundwater. Proper utilization of cotton stalk waste can address these issues. Hot mix asphalt (HMA) is essential for road construction, comprising heated aggregates and asphalt binder. However, the asphalt binder industry faces challenges due to dwindling crude oil reserves, causing limited availability and rising prices. Hence, there's an urgent need for enhancing existing asphalt binder performance and exploring alternative replacements^3–5^.

Researchers are increasingly interested in bio-oil modified binders, derived from waste or biomass pyrolysis, as alternatives to traditional asphalt binders, offering a sustainable and cost-effective solution^6–8^. In literature, the existing asphalt binder is partially or completely replaced with bio-oil modified binder extracted through pyrolysis. Bio-oil modified binder from swine manure that was extracted using the pyrolysis process was added. In that study, the extricated bio-oil modified binder was combined with different proportions (i.e., 10%, 5%, and 2% by mass of virgin binder) to alter the virgin binder and consequently its performance^9–13^. The Bending Beam Rheometer (BBR) and DSR were used to characterize the response over a wide temperature range, from high to low. The viscosity behaviour of modified specimens over a high temperature range was also assessed using a rotational viscometer (RV). Another study addressed the use of bio-oil modified binder produced by the pyrolysis of swine manure. 5% swine manure bio-oil modified binder was added to the virgin PG 64-22 binder^14^. The performance of the modified binder was assessed in both short- and long-term ageing conditions using the RV, DSR, and BBR. Oak wood was used to produce bio-oil modified binder, which was then preheated for approximately two hours^15,16^. In this study, bio-oil modified binder modified with 2% and 4% polyethylene content was used. The viscosity of the produced specimens at high temperatures was determined, and the effect of temperature on viscosity was evaluated.

Bio-oil modified binder was derived from diverse sources such as maize stalks, oak wood, shredded grass, and crumb rubber. These variants were utilized to modify both virgin binder and styrene–butadiene–styrene (SBS) incorporated binder^17,18^. Bio-oil modified binder from cedar sawdust, representing 9%, 6%, and 3% of the total binder mass, was incorporated into the specimens. These specimens were subsequently assessed under both ageing and non-ageing conditions, as well as at high temperatures^19^. High-temperature viscosity, density, and distress response were studied, leading to the development of a novel technique for extracting and utilizing bio-oil modified binder for modification^20–22^. The investigation involved evaluating the impact of varying temperatures and concentrations of bio-oil modified binder on viscosity. It found viscosity decreased notably with higher concentrations of bio-oil modified binder, especially below 135 °C. The research also included physical tests on the modified binder mixture^23^. The study examined asphalt properties through several tests and found bio-binder to be a promising alternative in asphalt technology, showing improved performance in various aspects. Increasing bio-oil modified binder content led to reduced penetration and enhanced ductile response, supporting its potential as a replacement for conventional asphalt technology^24^.

This study evaluates waste vegetable oil (WVO) and waste engine oil (WEO) as rejuvenating agents for asphalt binders with reclaimed asphalt pavement (RAP). Results show improved low-temperature properties and flexibility in RAP-containing asphalt mixes, indicating potential for enhanced performance in recycled asphalt applications^25^. The study investigates the impact of rejuvenation on RAP binders and mixes, exploring both short-term and long-term aging effects. Its objective is to evaluate the sustainability of pavement construction methods^26^. Laboratory experiments assessed Mahua oil's effectiveness in rejuvenating aged binder, exploring its potential as an asphalt binder rejuvenator to improve pavement performance and sustainability^27^.

Cotton stalk (CS) is defined as the waste derived from agricultural and industrial activities. The methods of open dumping and landfilling lead to the production of a significant amount of waste, which can lead to negative effects on the environment, including the contamination of subsurface water sources and a problem with land scarcity. This mass production of CS waste provides opportunity for recycling and its use for paving, which has never been tried in the form of bio-oil modified binder, to the best of the author’s knowledge. Therefore, this research’s aim is to characterize the pavement response of virgin binder and mixtures modified using bio-oil modified binder from CS.

## Objectives of the study

The primary objectives of this research are: (1) to evaluate the chemical, morphological, and rheological properties of base binder blended with bio-oil derived from agricultural waste; (2) to quantify the optimum dose of different bio-oils with the best performance; and (3) to produce an efficient, cost-effective, and environmentally friendly partially modified binder.

## Materials and methods

**Figure 1:**
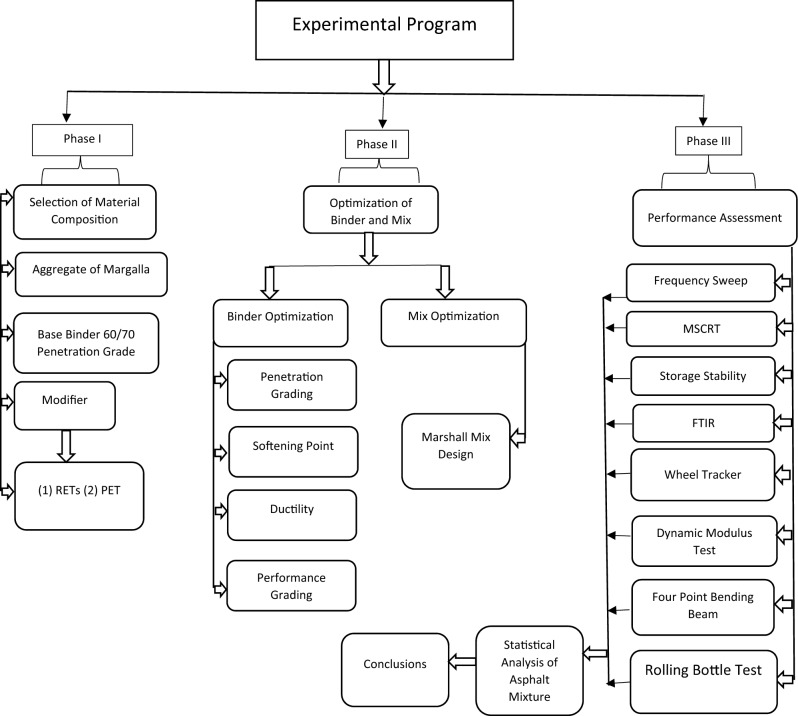
Flow chart of experimental methodology.

Figure 1 illustrates a schematic representation of the materials and methodology employed in this study to investigate the feasibility of using bio-oils as a partial substitute for virgin binder in asphalt mixtures. This study employed the fast pyrolysis method to extract bio-oil from CS, integrating it into conventional binder at concentrations of 5% and 10%. The experimental approach encompassed a thorough characterization of the CS bio-oil modified binders alongside the conventional binder, incorporating penetration, softening point, and ductility tests. Subsequently, the dynamic mechanical response of binder samples was analyzed using a dynamic shear rheometer (DSR). Following this, the modified binder was combined with aggregates to produce modified asphalt mixtures, which underwent rigorous testing for moisture sensitivity via the rolling bottle test (RBT) and the bitumen bond strength (BBS) test. Additionally, the performance of asphalt mixtures against rutting and fatigue cracking was evaluated using the dynamic modulus test, wheel tracker test (WTT), and four-point beam fatigue test (FPBFT). Comprehensive details regarding the materials, preparation process, and experimental program are shown below:

### Materials and preparation

#### Conventional asphalt binder

For this study, asphalt binder with a 60/70 penetration grade was sourced from Pak-Arab Refinery Limited (PARCO) in Pakistan. Table 1 outlines the specific characteristics of the PARCO asphalt binder.

**Table 1:** Properties of Conventional Asphalt Binder.

Properties	Standard Method	Specification Limits	Test Value
Penetration Test (0.1 mm, @25 °C)	AASHTO T49	60–70	66
Softening Point Test (°C)	AASHTO T53	46–56	50
Rotational Viscosity Test (Pa s, @135 °C)	AASHTO T316	≤ 3 Pa s	0.425
Ductility Test (cm, @25 °C)	AASHTO T51	100+	120
Specific Gravity Test	AASHTO T228	1.01–1.06	1.02

#### Cotton stalk

The cotton stalks (CS) utilized for the preparation of bio-oil modified binder were sourced from Faisalabad, Punjab, Pakistan. Bio-oil modified binder was procured from reputable suppliers using established methods to ensure both quality and quantity for experimental evaluation. Table 2 outlines the physical characteristics of CS^28^.

**Table 2:** Proximate analyses of cotton stalk^28^.

Elements	Test Result
Ash (wt%)	6.97
Moisture (wt%)	7.87
Fixed carbon (wt%)	15.62
Volatile (wt%)	69.54
Low heat value (MJ/kg)	15.99

#### Bio-oil

In this study, bio-oil was derived from fast pyrolysis of CS feedstock. Initially, the CS material underwent mechanical grinding to attain a particle size of approximately 0.5 mm. Preheating was then conducted to reduce moisture content before subjecting the CS to rapid heating, in the absence of air, at temperatures ranging from 450 to 600 °C^29^. The initial moisture content fell within the range of 10–20% by weight. During each run, a 30 g test sample underwent processing in the pyrolysis reactor for approximately 20 s, with temperatures varying from 450 to 600 °C. The variation in pyrolysis temperature was aimed at optimizing yield, resulting in an optimal yield of 53.3% achieved at 550 °C.

Rapid pyrolysis, involving the heating of CS and subsequent condensation of resulting vapors into a liquid state, was employed in this study to extract bio-oil. The residual biomass ash constitutes the remaining material at the bottom of the pile. To reduce the moisture in the CS, it is critical to sun-dry it first. Moisture content in control CS bio-oil was found to be between 6 and 8% by weight in the initial research. Table 3 shows the physical parameters of bio-oil derived from CS.

**Table 3:** Main properties and elements of CS boil oil^28^.

Principal Properties	Unit	CS Boil Oil
H_2_O	(wt%)	24.45
Lower heating value (LHV)	(MJ/kg)	17.78
pH	–	3.310
Viscosity	Ns m^−2^	125.1
Density	(kg/m^3^)	1160
S	(wt%)	0.210
O	(wt%)	49.50
N	(wt%)	0.305

#### Aggregates and gradation

**Figure 2:**
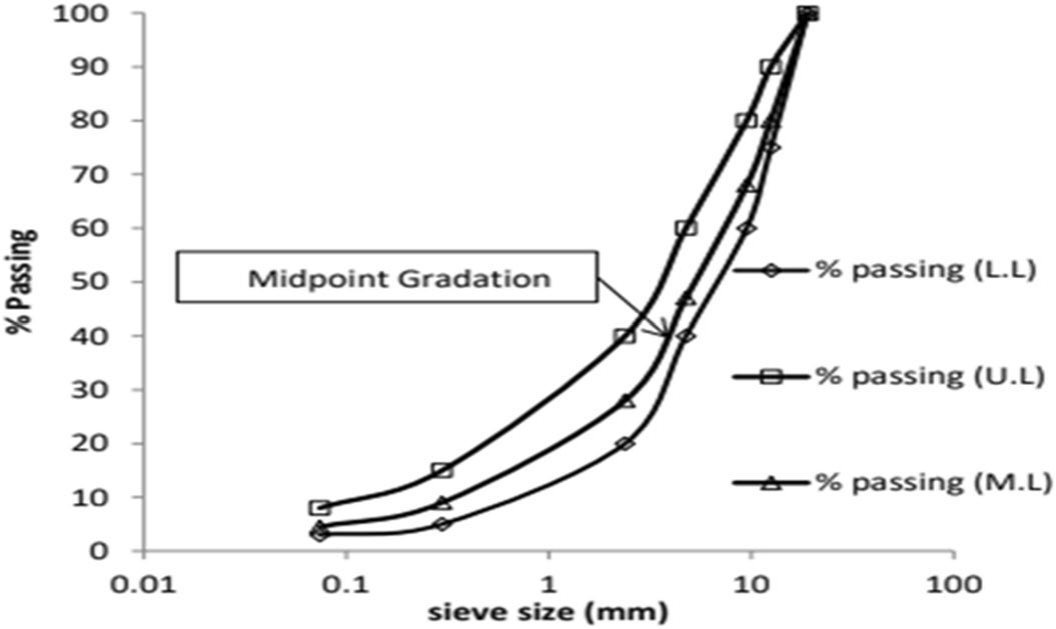
NHA Class B Gradation.

The Margalla region provided the source for the aggregates utilized in this investigation. Limestone makes up the aggregates found in Margalla, Pakistan^30^. Table 4 shows the physical properties of aggregate. This study utilized the class B aggregate gradation specified by the National Highway Authority (NHA), as illustrated in Fig. 2. Aggregates were cleaned and dried before the test.

**Table 4:** Material properties of Margalla aggregates.

Material Properties	Test Results (%)	Recommended Range by NHA* (%)^31^
Flakiness	5	10
Fractured particles	100	90 (min)
Elongation	11	10 (max)
Water absorption	1.02	2 (max)
Sand equivalent	75	50 (min)
Los Angeles abrasion	15	15 (max)
Soundness (Coarse)	7.1	8 (max)
Uncompacted voids	37.5	45% (min)
Soundness (Fine)	4.7	8% (max)

*National Highway Authority of Pakistan.

#### Process of preparing modified binder

The CS bio-oil modified binder was added to the base binder in the recommended proportions of 5% and 10% by weight. The selection of the proportions of CS bio-oil modified binder is based on a careful assessment and consideration of various factors, including prior research studies, preliminary experiments, and the desired objectives of the study. This study aims to determine the optimal dosage of CS bio-oil modified binder that would exhibit significant improvements in the performance characteristics of the base binder without compromising the overall quality and integrity of the asphalt mixture. To ensure uniform mixing of CS bio-oil modified binder at the specified percentages, a shear mixer was utilized in previous studies. The CS bio-oil modified binder and virgin binder were both heated to initial temperatures of about 120 and 60 °C, respectively. The materials underwent mixing at a temperature of 120 °C and a shear rate of 3000 rpm for a duration of one hour. Following these parameters consistently, three types of binder (two for CS bio-oil modified binders and one conventional binder) were used throughout this study.

#### Process of preparing modified asphalt mixture

The batched aggregate and asphalt binder were thoroughly mixed to prepare the asphalt mixture samples at the optimum binder content (OBC). The OBC of the control mixture sample, was determined to be 4.33% using the Job Mix Formula (JMF) method specified in ASTM D1559^32^. The specimens were then compacted by applying 75 blows for each face of sample, which was dropped from a height of 18 inches. All mixture samples were prepared with the same OBC to ensure consistency and facilitate better comparability of the test results^33^.

### Experimental program

#### Basic binder properties test

The assessment of the asphalt binder's consistency is conducted through a penetration test. Both bio-oil modified binder and control (unmodified) samples were tested under defined conditions based on AASHTO T49^34^. The softening point of the produced specimens was determined using the ring and ball apparatus in accordance with ASTM D36 standard^35^. A ductility test was performed based on ASTM D113^36^ on every produced sample with the use of a ductilometer.

1 $$\text{PI} = \frac{\text{20-500A}}{\text{1+50A}}$$

2 $$\text{A}=\frac{\text{log}\left(800\right)-\text{log}(\text{Pen at T})}{\text{SP}-25}$$

whereas, PI is the penetration index, Pent at T represents the penetration value of the asphalt binder at a specific temperature T and SP is the softening point of the asphalt binder.

#### Rheological properties tests

##### Performance grading (PG) and frequency sweep tests

Frequency sweep and PG grade tests were carried out to evaluate viscoelastic response in the linear range using DSR based on AASHTO T-315^37^. The frequency sweep under the strain-controlled approach was chosen for the wide range of temperatures (22, 34, 46, 58, 70, and 82 °C)^38^. Additionally, frequencies between 0.1 and 10 Hz were used for each temperature. In agreement with the literature, the strain levels exerted were 2% and 10% for virgin and CS-oil binder, respectively^38^. The 8 mm and 25 mm plate geometries were chosen for higher (> 46 °C) and lower (46 °C) temperatures, respectively, with a gap of 2 mm and 1mm^39^. In addition, a PG-grade test was conducted using a 25 mm plate geometry and a frequency of 10 Hz. The point where G*/sinδ came below 1 kPa corresponded to failure temperatures^38^. In Eq. 3, the sigmoidal equation is stated.

3 $${\text{Log}}\left| {\text{A}} \right| = \updelta + \frac{\upalpha }{{1 + {\text{e}}^{{\upbeta + \upgamma \log \left( {{\text{f}}_{{\text{r}}} } \right)}} }}$$

where, A is the complex modulus, δ is the phase angle, *f*_*r*_ is the reduced frequency, α is the difference between the minimum and maximum G*, γ and β are the shape factors^40^.

##### Multi stress creep recovery test

Both the conventional and bio-oil modified binder modified asphalt binder were subjected to a multiple stress creep recovery (MSCR) test using DSR in accordance with AASHTO T315^37^. The viscoelastic nature of Asphalt binder is a widely acknowledged phenomenon. At moderate temperatures, the behavior of asphalt binder is viscoelastic, with the dominant behavior being viscous at high temperatures and elastic at low temperatures. When constant stress (creep) is applied to Asphalt binder, strain is produced in the asphalt binder, and after the stress is relieved, the Asphalt binder tends to return to its original form, but some residual strain (permeant strain) will remain in the Asphalt binder, whereas an elastic strain is recovered after the load is removed. To assess the permanent deformation resistance of asphalt binder under varying stress levels ranging from 0.25 to 3.2 kPa and temperature ranges between 58 and 76 °C, a creep compliance test was conducted. This test aimed to cover all the PG ranges of samples and to evaluate the elastic response of asphalt binder. Non-recoverable creep compliance (Jnr) was measured to determine the asphalt binder's permanent deformation resistance to repeated creep loads. After applying a one-second creep load, a recovery period of nine seconds was given to the sample. The residual strain in the specimen in terms of Jnr after the creep and recovery times was calculated using Eq. (4).

4 $$\text{Jnr }=\text{Avg}. \frac{\gamma \mu }{\tau}$$

where;Jnr = permanent strain in a binder after creep implementation in kPa^−1^^41^, $\gamma \mu$ = permanent strain after 9-s interval, $\tau$ = shear stress applied for 1-s interval.

Through this test, the stress sensitivity of modified and unmodified samples under different loading conditions was determined. Anton Paar DSR was used in this study to evaluate the rheological properties of asphalt binder. For high temperature 25 mm diameter plates were used while for low temperature the 8 mm diameter geometry were used. Gap between two parallel pates were 1 mm and 2 mm for 25 mm and 8 mm respectively.

#### Asphalt binder bond strength test

**Figure 3:**
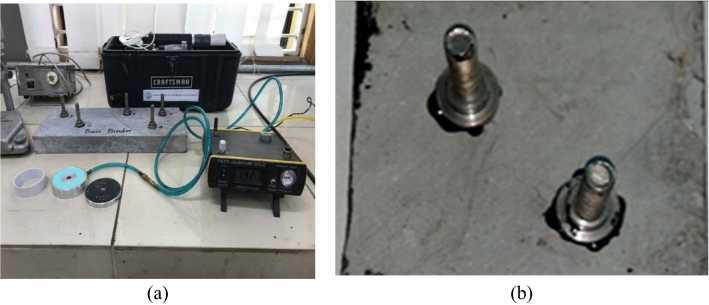
Asphalt binder Bond Strength Test (**a**) PATTI Instrument (**b**) Sample Prepared for BBS.

The asphalt binder bond strength (BBS) test was conducted to measure the tensile force required to detach the stub from the base plate, in the presence of binders as shown in Fig. 3. The test was conducted following the controlled conditions outlined in ASTM D4541^42^. Initially, a 15" × 6" × 1.5" base plate was prepared by trimming a Margalla limestone slab. The base plate was cleaned for one hour at 60 °C using an ultrasonic cleaner. Prior to the test, the base plate was preheated in an oven at 150 °C for 60 min and then reheated for 30 min at 75 °C. The stubs were also heated for 30 min at 65 °C. A pressure of 120 psi was applied to the cylinder, and 0.4 g of either virgin or modified binder was applied to the stubs and pressed onto the base plate of aggregates. Excess asphalt binder was carefully removed from all samples using a cutter. Specimens were conditioned as either unconditioned (dry) or moist conditioning specimens after 24 h. The F4 piston and circular spacer were carefully set, and the pressure was gradually applied to the stubs during the test. The pull-off test strength (POTS) values were recorded for each specimen. Equation 5 was used to estimate POTS^43^.

5 $$POTS= \frac{\left(BP*Ag\right)-C}{Aps}$$

whereas, BP = Burst Pressure, C = Piston Constant (C = 0.286 in^2^), Ag = Contact Area of Gasket (Ag = 4.06 in^2^), Aps = Pull-out Stub Area (Aps = 0.193 in^2^).

#### Rolling bottle test

**Figure 4:**
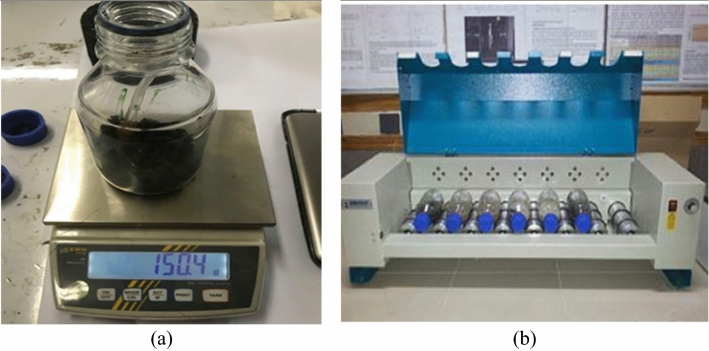
Rolling Bottle Test (**a**) Moisture susceptibility Apparatus (**b**) Rolling Bottle Sample.

The moisture damage to each material was evaluated using the rolling bottle test (RBT) (EN 12697-11A)^44^. After being coated with virgin or modified binder, the coated aggregates were mechanically abraded by water as shown in Fig. 4. After being completely coated with virgin or modified binder (weight: 8 gm), aggregates (weight: 170 gm) were put in bottles (500 ml) of deionized water with a stirrer. Then, the bottles were rotated at 60 RPM for interaction. The results in the form of the remaining coating percentage were noted at diverse time intervals (6 h, 24 h, 48 h, and 72 h). In this hypothetical scenario, the key indicators might include the ability of the asphalt mix to resist deformation and maintain structural integrity under simulated rolling conditions. Observations would focus on signs of rutting or other distresses, providing insights into the mix's performance and stability under the stresses encountered in a rolling environment, such as those experienced by vehicles on a roadway.

#### Assessment on the asphalt mixture performance

##### Wheel tracking test

The resistance of the produced mixes (virgin or modified) against permanent deformation was evaluated referring to BS EN 12697-25^45^. The test was run using a 700-N wheel that could complete 53 passes in 60 s. The selected protocols were 12.5 mm and 50 °C (rut-depth criteria). These methods were chosen to assess the mixtures' resistance to permanent deformation in Pakistan's predominately hot climate^46^. The slabs with the specified dimensions (300 mm × 300 mm × 50 mm) were first prepared by compaction using the roller compactor to conduct the stated experiment. The produced slabs were then put into the frame of the apparatus for testing while being passed by wheels frequently. The device was programmed to automatically stop at 10,000-wheel passes when the test was completed. The use of a software program on the PC linked with the apparatus allowed the rut depth to be automatically registered for each produced sample.

##### Dynamic modulus test

After adding CS bio-oil modified binder to the produced mixes, the stiffness effect was assessed using the Dynamic Modulus Test, AASHTO TP62^47^. Cylindrical specimens with dimensions of 150 mm diameter and 170 mm height, along with an air-void content of 5.5 ± 0.5%, were prepared using the Superpave gyratory compactor. Afterwards, these samples were subjected to coring and cutting into new dimensions (101.6 mm dia. and 150 mm height). At 4.4, 21.1, 37.5, and 54.4 °C and 25, 10, 5, 1, and 0.5 Hz, all the produced samples were evaluated^48^. The output from the examination includes values of dynamic modulus at formerly stated conditions. The sinusoidal loads of about 53 kPa, 195 kPa, 525 kPa, and 1050 kPa were applied to the produced samples within the equipment frame.

##### Four-point beam fatigue test

The fatigue resistance of the produced mixes, both virgin and modified, was evaluated using the four-point beam fatigue test, AASHTO T321^49^. The stress-control mode was used to conduct the test. Firstly, the stress-controlled mode allows for a more accurate and controlled application of stress levels throughout the test. This helps in simulating the actual stress conditions that the material would experience in real-world applications, such as the repeated loading of a structural component. Secondly, stress-controlled testing is particularly relevant when the material being tested exhibits significant strain hardening or strain softening behaviour. In such cases, controlling the stress levels allows for a more consistent and controlled response from the material, ensuring that the test results are representative of its true fatigue behaviour. The selected protocols for this test were 10 Hz (frequency level) and 20 °C (set temperature). The beams produced were 380 mm 50 mm 63 mm in size. The produced beams passed repeated loading tests. The test was programmed to automatically end after the initial stiffness of the produced mixes had decreased by 50%.

#### Statistical analysis

Statistical analysis was conducted using the SPSS software to further evaluate the test results. This analysis can be useful in determining the extent to which CS bio-oil modified binder affects the asphalt binder and mixes. The asphalt binder and mixture were shown to be significantly impacted by CS bio-oil modified binder. The recorded laboratory data was further analyse using variance analysis (ANOVA) for this analysis with a 95% confidence level, the general linear model was selected. The dependent variable for each test was chosen as the result of the corresponding test (with 3 replicates). However, the independent variables varied with each test type (as presented in Table 5). The *P*-values from the statistical analysis were then compared with α = 0.05 (also known as reliability) to determine whether an effect was significant. According to the specified criteria, the null hypothesis should be accepted if *P* < 0.05. The independent variable does not significantly affect the dependent variable, according to the null hypothesis. The null hypothesis in this instance also stated that "One independent variable does not have an interaction effect on the other independent variable, and vice versa." This evaluation was done for RBT and BBS”.

**Table 5:** Variables opted for each test (statistical analysis).

Test type	Dependent variable	Independent variable(s)
Four-point beam fatigue test	Failure cycles	Bio-oil modified binder Percentage
Rolling bottle test	Coating (%)	Bio-oil modified binder Percentage, time interval
Asphalt binder bond strength test	POTS	Bio-oil modified binder Percentage, condition type
Cooper wheel tracker test	Rut depth	Bio-oil modified binder Percentage
Dynamic modulus test	Dynamic modulus	Bio-oil modified binder Percentage

## Results and discussion

### Basic properties of asphalt binders and thermal susceptibility

**Figure 5:**
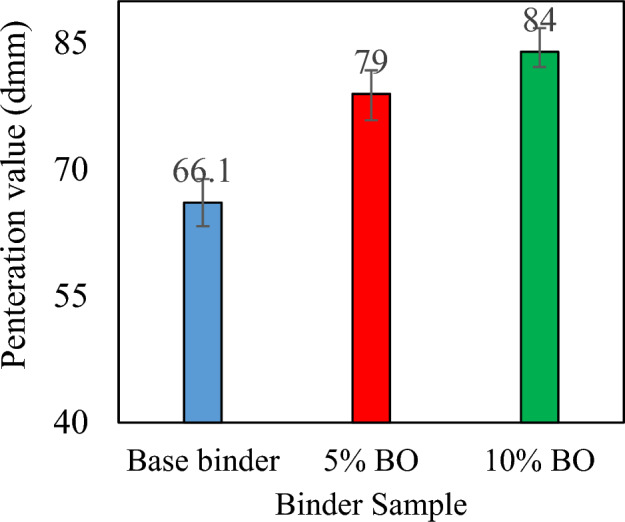
Penetration values for each sample.

This section presents an analysis of the consistency test results in terms of penetration, ductility, softening point, and penetration index. The increasing penetration trend is commonly accompanied by an increase in the quantity of CS bio-oil modified binder, which denotes the start of the softening-gain mechanism, as shown in Fig. 5.

**Figure 6:**
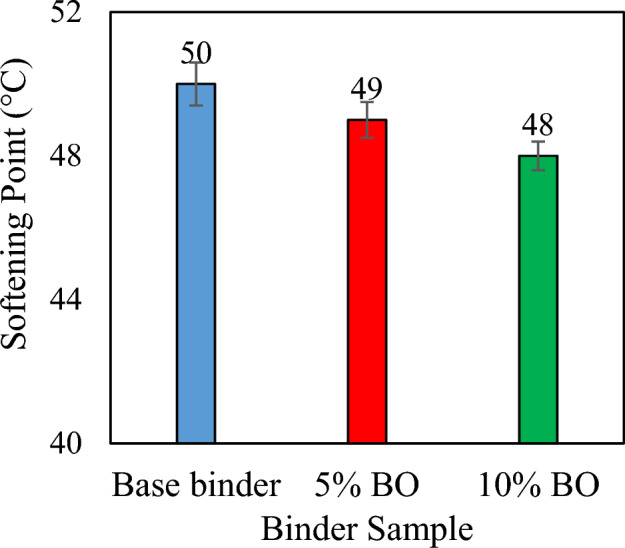
Softening point values for each sample.

In comparison to the virgin binder, a percentage rise of approximately 27.1% and 19.5% is observed for penetration values of 10% and 5% CS bio-oil modified binder, respectively in Fig. 5, the rising trend for penetration is typically seen with increasing CS bio-oil modified binder content, which indicates the initiation of the softening-gain mechanism. In comparison to the virgin binder, a percentage rise of approximately 27.1% and 19.5% is observed for penetration values at 10% and 5% CS bio-oil modified binder, respectively. This trend is confirmed by the softening point trend (Fig. 6), which also shows the production of soft binder by increasing the amount of CS bio-oil modified binder. Compared to the virgin binder, the lowest softening point was observed at a concentration of 10% CS bio-oil modified binder. However, the lower softening point values with increasing CS bio-oil modified binder content suggest a rise in thermal susceptibility^50^, which will be further evaluated through the PI values at the end of this section.

Table 6 presents the findings of the ductility test conducted on the virgin and modified binders. Additionally, Table 6 indicates that the rise in ductile response was mainly due to the higher CS bio-oil modified binder content in the virgin binder, which is linked to the softness-gain mechanism. The highest ductile response was observed when 10% of CS bio-oil modified binder was combined with the virgin binder, resulting in the greatest increase in ductility compared to the virgin binder. The impact of CS bio-oil modified binder on the ductility of binder blends was statistically significant. Moreover, maximum ductility is an indication of better flexibility as well as resistance to fracture. The incorporation of CS bio-oil modified binder at 10% and 5% weight percentages resulted in a change in the penetration grade of the virgin binder, to Pen-grade 80/100 and Pen-grade 70/80, respectively. The incorporation of CS bio-oil modified binder resulted in the production of softer binders. Furthermore, the ductility, penetration, and softening point values of CS bio-oil modified binder fall within the typical paving grade specifications used in Pakistan (refer to Table 6)^51^. Moreover, the production of softer binders indicates the potential of CS bio-oil modified binder to soften hard binders, highlighting its applicability for recycling materials^52^.

**Table 6:** Ductility Values for Each Sample of Binder.

Samples	Ductility Value (mm)
Base binder	100+
5%BO	115+
10%BO	120+

**Figure 7:**
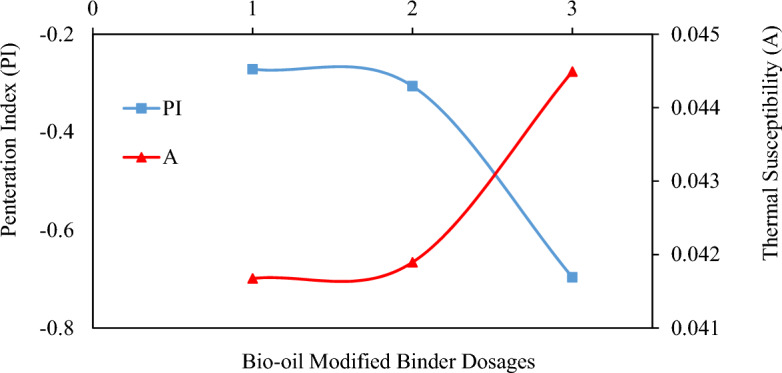
Relationship between Penetration Index and Thermal Susceptibility of Tested Asphalt binder.

The Penetration Index (PI) measurement offers quantitative insights into the thermal susceptibility of materials^53^. The Penetration Index (PI) values typically fall within the range of − 3 to + 7, indicating the high or low thermal susceptibilities of asphalt binders. Equations (2) and (3) were employed to calculate the PI values and thermal susceptibility (A), following the methodology outlined in the literature^54^. Figure 7 shows the results of asphalt binder sample penetration index. The analysis of the results shows that increasing the CS bio-oil modified binder content resulted in a decrease in PI values. The 10% CS bio-oil modified binder content in virgin binder was used to determine the bottom PI value. The decreasing PI values and increasing trend of susceptibility (A) values demonstrate that the addition of CS bio-oil modified binder increased the susceptibility of the binders treated with it. However, the PI values remain within the permissible limits defined by the earlier studies^33^ as an allowable limit for paving binder (− 2 to + 2). The CS bio-oil modified binder binders are therefore inferred to be suitable for use in paving applications, as previously expected. Yet, the primary problem with predicting thermal susceptibility from PI estimation is that, when calculating A values, only two temperatures are used (i.e., 20 °C and 25 °C for softening point and penetration respectively). Because of this, there is a need to study the rheology of virgin and modified binders at wide temperatures and frequency levels, for which testing through DSR is the best option.

### Rheological behaviour

**Figure 8:**
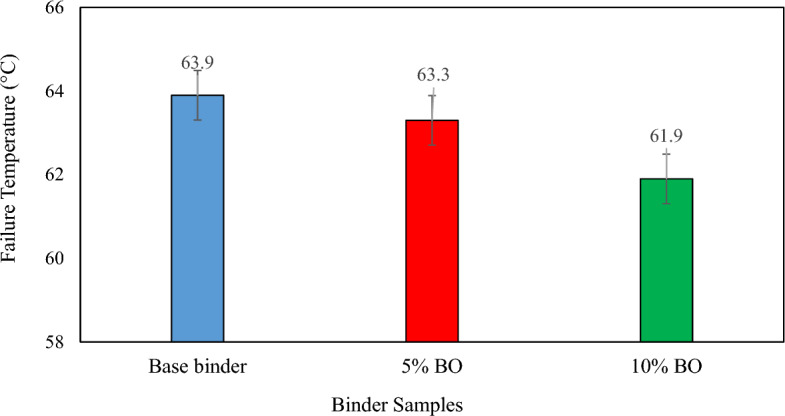
Failure temperature of tested binder samples.

The rheological assessment of the asphalt binders and mixtures was performed to understand their flow and deformation behaviour. The results indicated that the addition of bio-oil modified binder in the binder led to notable changes in its rheological properties as shown in Fig. 8. The rheological assessment demonstrated that adding bio-oil modified binder reduced the viscosity of the binder, enhancing its resistance to flow at low temperatures. While this may increase susceptibility to rutting, it also improves flexibility by decreasing stiffness, reducing the risk of cracking due to thermal changes. These findings offer critical insights into the impact of bio-oil modified binder on asphalt mixtures, guiding the development of more durable and sustainable road construction materials.

#### Performance grading (PG) of bio-oil modified binder-modified asphalt binder

**Figure 9:**
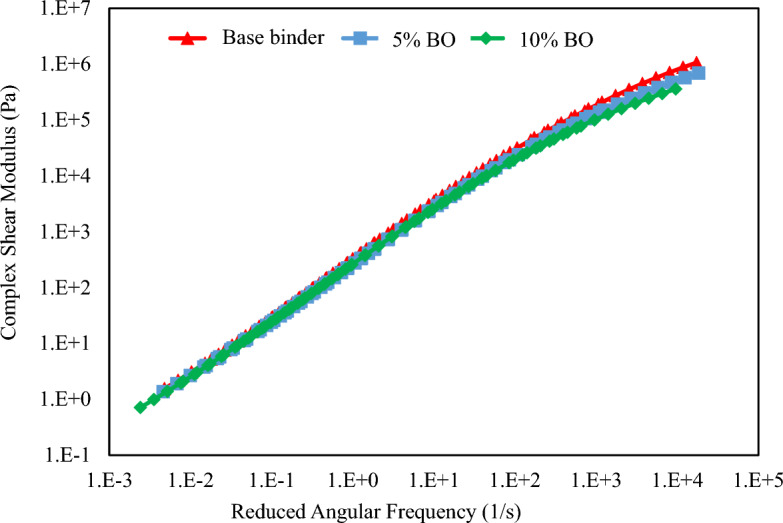
Master Curves of Reduced Angular Frequency versus Complex Shear Modulus.

Figure 9 shows the results of the PG-grade test, indicating failure temperatures. Overall, adding CS bio-oil modified binder reduced the failure temperatures compared to using base binder. When compared to virgin binder, which has a failure temperature of 63.9 °C, the most significant decrease in failure temperature is seen at 10% content of CS bio-oil modified binder. Additionally, any binder specimen can be selected for PG-grade using the failure temperatures. Startlingly, the PG-58 was similar for every binder specimen. Thus, as far as its practical implications are concerned, the binders modified with CS bio-oil modified binder can be used as surrogates for a virgin binder, which is additionally confirmed by the rheological behaviour studied previously, as there was equal rut resistance and improved fatigue resistance.

**Figure 10:**
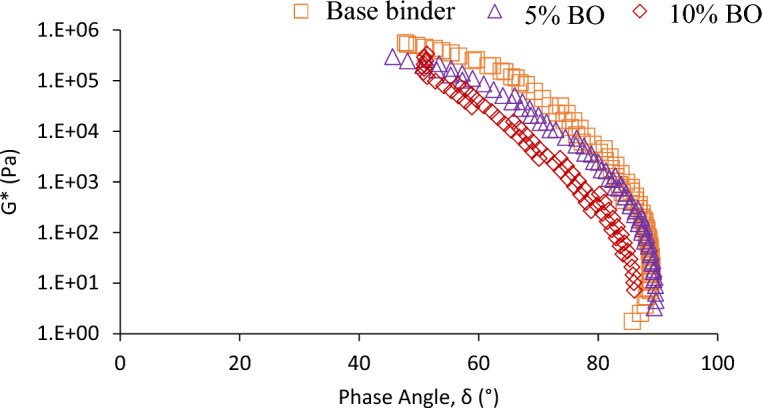
Effects of Bio-oil Content on Bitumen Viscoelastic Properties.

The black diagram, independent of frequency and temperature, provides a comprehensive overview of the data in a single graph. It may be noted from the Fig. 10 that the viscoelastic properties of bitumen are very sensitive to CS bio-oil content as there is a significant change in path of the unmodified binder and binder modified with different dosages of CS bio-oil. Moreover, an increase in temperature correlated with a rise in the phase angle of bitumen and a decrease in G* value. Notably, beyond a certain threshold depicted in Fig. 10, the phase angle of 5% and 10% CS bio-oil demonstrates a reduction at high temperatures and low frequencies compared to the base binder, indicating heightened elastic behavior of bitumen for these proportions. Consequently, the addition of CS bio-oil enhances resistance against deformation in bitumen.

The section represents a rheological assessment as well as the statistical examination of the results acquired through DSR (frequency sweep and PG-grade tests) to study the impact of adding CS bio-oil modified binder in virgin or modified binder at various temperatures and frequencies. The rheological assessment comprises (a) the complex modulus (G*), (b) fatigue factor (G*.sinδ), (c) rut factor (G*.sinδ), and (d) PG-grading. The master curves in Fig. 9 are comprised of reduced frequency (*fr*) values along the x-axis and complex modulus (G*) values along the y-axis. They were produced using a sigmoidal function at a reference temperature of 58 °C. Referring to master curves for both the virgin binders and CS bio-oil modified binder, it was observed that the complex modulus values in the high-temperature range reduced as the proportion of CS bio-oil modified binder increased, with the most substantial reduction noted at a 10% CS bio-oil modified binder concentration. This indicates that CS bio-oil modified binder has a greater potential to enhance the fatigue resistance of virgin binder due to the softening-gain mechanism at low temperatures. Moreover, the complex modulus values for each CS bio-oil modified binder specimen just slightly change, demonstrating nearly similar high-temperature performance for both virgin and modified binders. This shows that the CS bio-oil modified binder specimen can reduce stresses and withstand increased external loading just like a virgin binder. Additionally, these results are consistent with other studies on bio-oil modified binder^40^. A statistical analysis of the data in Table 7 was done to corroborate these trends. The null hypothesis is supported by enough evidence when the *P*-value is greater than α = 0.05. Thus, it can be confirmed that there was nearly equal performance between CS bio-oil modified binder binders and virgin binders. Additionally, it confirms earlier findings that the addition of any amount of CS bio-oil modified binder had no effect on virgin binder performance at high temperatures.

**Table 7:** Statistical analysis of dynamic modulus results.

Source	DF	F	*P*-value	Significant Effect
Corrected Model	2	1.005	0.321	
Intercept	1	1072.871	0.000	
Bio-oil modified binder percentage	2	1.005	0.321	No

**Figure 11:**
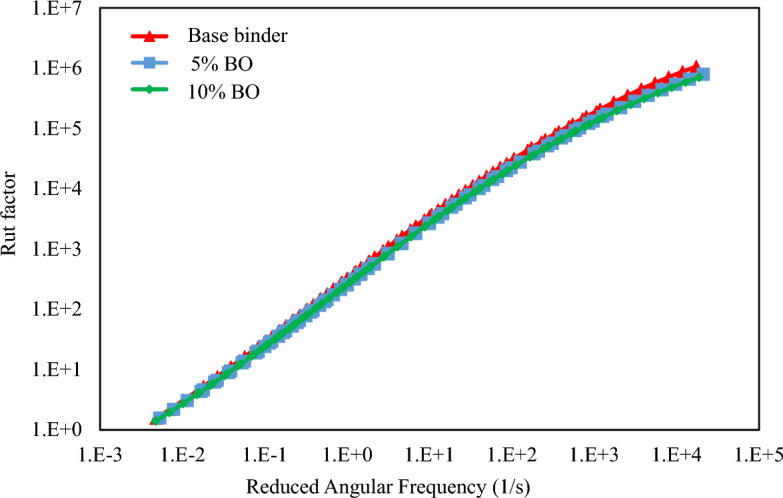
Variation of Rut Factor with Reduced Frequency.

For a more effective view of rheological behaviour, the Superpave factors (fatigue and rut factors) were also estimated. By considering these factors, it is possible to better understand how virgin and modified binders behave rheologically in terms of fatigue and rutting resistance^55^. For each temperature (82 °C, 70 °C, 58 °C, and 46 °C), G*/sin (δ) (rut factor) was calculated at a frequency of 10 rad/s, in accordance with recommendations from the prior study^56^. The fatigue factor, G*.sin (δ), was determined in a similar way to this at two distinct temperatures, 34 and 22 °C. Figure 11 shows temperatures along the x-axis and values for G*/sin (δ) along the y-axis. The G*/sin (δ) is slightly affected by the addition of CS bio-oil modified binder contents, as expected, showing that both virgin and modified specimens have equal rut resistance in the high-temperature range. These results confirm the previous assessment of rheological behaviour studied through master curves of complex modulus.

#### Asphalt binder's sensitivity to stress variations

**Figure 12:**
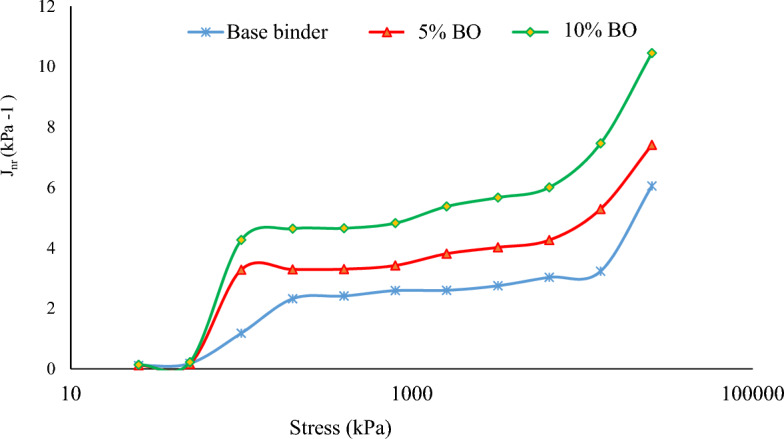
Stress (kPa) and Jnr (kPa^−1^) Values for Each Sample.

Using a loading mode that approximated real pavement loading, the MSCR test data were analysed to simulate real pavement force and deformation. This represents how the materials behave in hot environments. For an asphalt binder to meet the requirements of high quality, it is crucial that it demonstrates sufficient elasticity and recovery capabilities over a broad range of high temperatures, while also being able to resist continuous deformation under elevated temperature conditions. The non-recoverable creep compliance (Jnr) was measured at 64 °C, with stress as the independent variable and percent recovery as the dependent variable, as depicted in Fig. 12.

While the recovery rate decreases as stress increases, the value of Jnr increases along with it, with the amount varying according to the type of binder. The previous study indicates that stiffer binder Jnr values are lower than softer binder Jnr values. Figure 12 illustrates how a stiffer grade binder can be converted into a softer one by adding bio-oil modified binder, with the Jnr values increasing as a result. Figure 12 demonstrates how the addition of bio-oil modified binder affects the rheological properties of asphalt binders. It shows the relationship between the stiffness of the binder, represented by the Jnr values, and the addition of bio-oil modified binder. In the figure, as move from left to right along the x-axis, increasing the amount of bio-oil modified binder added to the original asphalt binder. As a result, the stiffness of the binder decreases, which is reflected in the increasing Jnr values along the y-axis. This trend indicates that the addition of bio-oil modified binder softens the original asphalt binder, making it less stiff. This is important because it demonstrates how the properties of asphalt binders can be modified by incorporating additives like bio-oil to achieve desired performance characteristics, such as improved flexibility or reduced susceptibility to cracking.

### Moisture susceptibility

Moisture can be a significant factor in causing premature failure of asphalt pavements, as it can decrease both the strength and stiffness of the material, leading to various types of distress, such as ravelling and hydraulic damage. This section investigates into the effects of incorporating CS bio-oil modified binder into both base and modified binders, particularly regarding their susceptibility to moisture. The results of the BBS and RBT tests are analysed to evaluate the response of the mixtures to damage caused by moisture. The BBS and RBT test results are analysed to evaluate the mixtures' susceptibility to moisture damage and their ability to withstand such effects.

#### Bitumen-aggregate adhesion (BBS results)

In this section, the pull-out tensile strength (POTS) values obtained from the BBS results were analysed to evaluate the adhesive strength between unmodified or modified binders and aggregates.

**Figure 13:**
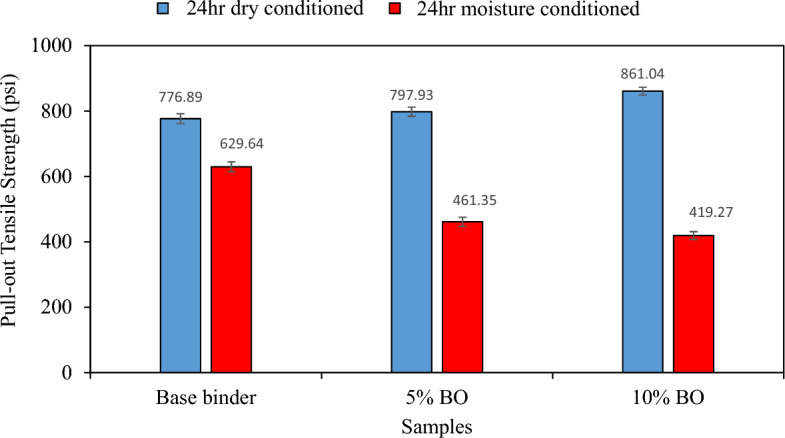
Bond strength between aggregates and binder.

The results presented in Fig. 13 exhibit a notable enhancement in the bond strength between the binder and aggregates with the increasing concentration of CS bio-oil modified binder in dry conditions. The highest POTS value recorded at 10% CS Bio-oil modified binder content signifies a substantial improvement in the bond strength between the aggregates and binder in the absence of moisture. The wettability concept, which is defined as the covering of aggregates with binder, can be used to describe the philosophy behind these results. Usually, soft binders possess wettability when compared to hard binders. More CS bio-oil modified binder was added, and as a result of the softness-gain mechanism, this promoted improved wettability, which in turn increased POTS values in dry conditions^57,58^. It is a well-known fact that binders with high wettability are more susceptible to the negative effects of stripping and emulsification in the presence of water. This is primarily attributed to the increased number of vulnerable sites on the aggregate surface that result from high wettability, which leads to enhanced water permeability^57,58^. This is supported by a decrease in POTS under moist conditions when more CS bio-oil modified binder content is added to virgin binder, demonstrating the severe action of water as a result of the softness-gain mechanism^59^. The highest reduction, which is seen for 10% of the CS bio-oil modified binder concentration, indicates a weaker binding between the binder and aggregate. Table 8 presents the statistical analysis results in more detail to determine the significance of the CS bio-oil modified binder percentage and test condition. The fact that the null hypothesis was rejected shows that the condition type and CS bio-oil modified binder, respectively, have a considerable impact on the bond strength of asphalt binder and aggregates. Additionally, the percentage of CS bio-oil modified binder and the condition type (either moist or dry) interact significantly (null hypothesis is rejected, *P* 0.05). This demonstrates the interaction of the effects of condition type and CS% level on the bond strength between asphalt binder and aggregate.

**Table 8:** Statistical analysis of BBS test results.

Source	df	F	*P*-value	Significant effect
Corrected Model	5	256.831	< 0.001	
Intercept	1	19,434.061	< 0.001	
Bio-oil modified binder percentage	2	23.574	< 0.001	Yes
Condition	1	1069.531	< 0.001	Yes
Bio-oil modified binder percentage * Condition	2	83.739	< 0.001	Yes

#### Remaining coating around aggregates in moist condition (RBT results)

**Figure 14:**
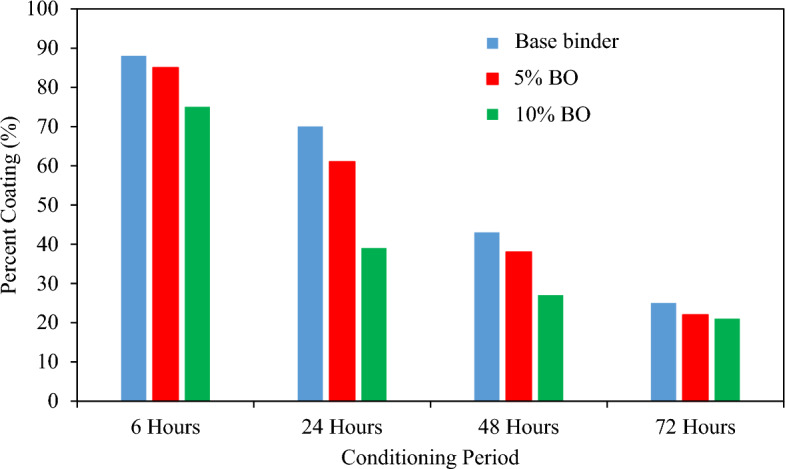
Coating Level for Base Binder and the CS modified Bio-binders.

Generally, the coating of binder around a single aggregate was reduced at various time intervals that resulted in a maximum decrease at 10% CS bio-oil modified binder concentration (Fig. 14). The study findings indicate that the bond strength between binder and aggregates is highly influenced by the addition of CS bio-oil modified binder, and this effect is further enhanced in the presence of water due to the increased content of bio-oil modified binder. These observations support the hypothesis that increased wettability of the binder leads to a higher probability of binder emulsification and stripping. The statistical analysis of the RBT results presented in Table 9 supports these conclusions, showing a significant impact of both the time interval and % of CS bio-oil modified binder on the bond strength between the binder and aggregates. The *P*-values for both variables were less than α = 0.05, leading to the rejection of the null hypothesis. There is no interactive influence between the percentage of CS bio-oil modified binder and the other variable, time interval, as shown by the *P*-value for the case "CS bio-oil modified binder x time interval" being greater than α = 0.05. This shows the independent influences of time and the amount of CS bio-oil modified binder on the moisture sensitivity of materials. Further supporting the damaging effect of moisture on the strength of the binding between binder and aggregate are BBS data that demonstrate the addition of CS bio-oil modified binder improves the CS bio-oil modified binder binder's moisture sensitivity. The damage caused by moisture is controlled using various modifiers, which include hydrated lime, Portland cement, and polymers (in liquid form). Further research is warranted to explore methods for improving the moisture resistance of CS bio-oil modified binder binders. The statistical analysis presented in Table 9 reveals that both the percentage of CS bio-oil modified binder and the time interval have a statistically significant impact on the bond between aggregates and asphalt binder. The impacts of both parameters on moisture resistance are interrelated, as evidenced by a comparable interactive effect between time interval and CS bio-oil modified binder content. The modified binder's reduced capacity to withstand moisture is caused by the addition of CS bio-oil modified binder, and this result is consistent with prior BBS test results.

**Table 9:** Statistical Analysis of Rolling Bottle Test (RBT) Results.

Source	df	F	*P*-value	Significant effect
Corrected Model	11	470.795	< 0.001	
Intercept	1	22,052.250	< 0.001	
Bio-oil modified binder percentage	2	201.000	< 0.001	Yes
Time	3	1540.250	< 0.001	Yes
Bio-oil modified binder percentage * Time	6	26.000	< 0.001	Yes

### Asphalt mixture testing results

The durability of a pavement, which is closely tied to its performance, is a key factor in estimating its structural capacity^60^. The use of additives to improve the performance of asphalt mixtures can result in longer pavement service life and reduced maintenance costs, as well as improved resource conservation, particularly in terms of aggregates and binder. The resistance of pavement against moisture damage, rutting, and wear plays a key role in determining its durability. This section presents a comparison of the performance of asphalt mixtures containing CS bio-oil modified binder additives and virgin mixtures.

#### Wheel tracker test

**Figure 15:**
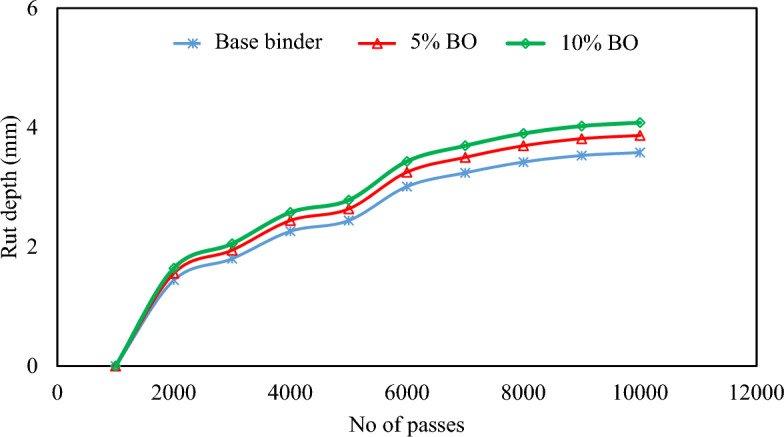
Rut depths at consecutive and cumulative passes till 10,000 for virgin/modified mixtures.

This results of the Wheel Tracker Test (WTT) in Fig. 15. The test is specifically designed for precisely evaluating resistance to rutting in the form of rut depth, to further support the analysis performed in the preceding section. The outcomes acquired through the WTT test are graphically shown in Fig. 15 (rut depths at consecutive and cumulative passes till 10,000 for virgin and modified mixtures). Generally, cumulative passes with more CS bio-oil modified binder content show a decrease in rut depth. In addition, the rut depths at initial wheel passes for each base or modified mixture have a sharp inclination (sharp increase), which later changes to a slow and gradual rise. The actual causes behind this outcome are material densification and aggregate locking under the repeated wheel loads. The rut depth consequently abruptly increases at the beginning of the WTT^61^. After 10,000-wheel passes, the rut depths of both the virgin and CS bio-oil modified binder modified mixtures were measured and compared. The analysis revealed that the rutting resistance of the mixtures did not improve significantly with an increase in CS bio-oil modified binder content. In comparison to the virgin mixture, there is a percentage increase of 5.8% and 3.1% for the contents of 10% and 5% CS bio-oil modified binder, respectively. This supports the previous claims of equal rut resistance in virgin and modified mixtures. The statistical analysis in Table 10 was conducted to determine whether the observed improvement in rutting resistance with increasing CS bio-oil modified binder content is statistically significant. After comparing the final rut depths following 10,000-wheel passes, the results showed no statistically significant increase in the pavement's ability to resist rutting with the addition of CS bio-oil modified binder (*P*-value > 0.05).

**Table 10:** Statistical Analysis of Rut Depth in modified binder.

Source	df	F	*P*-value	Significant effect
Corrected Model	2	1.352	0.327	
Intercept	1	3469.210	< 0.001	
Bio-oil modified binder percentage	2	1.352	0.327	No

#### Dynamic *modulus* test

**Figure 16:**
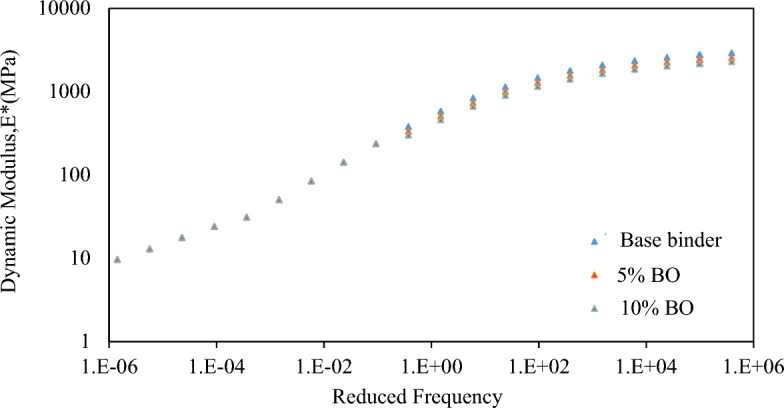
Dynamic modulus master curves for virgin/modified mixtures.

This section compares the results of the virgin mixtures with those of the CS bio-oil modified binder modified mixtures, with a focus on the dynamic modulus, which is an essential design parameter for flexible pavements. The impact of dynamic modulus on pavement performance, specifically in terms of fatigue and rutting resistance, is also analysed. The dynamic modulus master curves for both the virgin and modified mixes are illustrated in Fig. 16. In agreement with the previous finding, the softening-gain process causes an obvious decrease in E* values for higher concentrations of the CS bio-oil modified binder mixture at low temperatures. The maximum decrease is noted at 10% content of CS bio-oil modified binder, which shows better resistance of the material against fatigue when compared to a virgin mixture^62^. The statistical analysis described above further confirms these results. Furthermore, as expected, a negligible increase in E* values are observed at low frequencies, indicating that CS bio-oil modified binder mixtures have the same response to distress (rutting) as virgin mixtures.

Table 11 presents the results of statistical analysis of dynamic modulus in the high-frequency range, which confirms that adding CS bio-oil modified binder can enhance the mixture's resistance to fatigue cracking (the null hypothesis is rejected as the p-value is less than 0.05).

**Table 11:** Statistical Analysis of Dynamic Modulus Results of modified binder.

Source	df	F	*P*-value	Significant effect
Corrected Model	2	28.643	0.001	
Intercept	1	6060.623	< 0.001	
Bio-oil modified binder percentage	2	28.643	0.001	Yes

#### Four-point beam fatigue test

**Figure 17:**
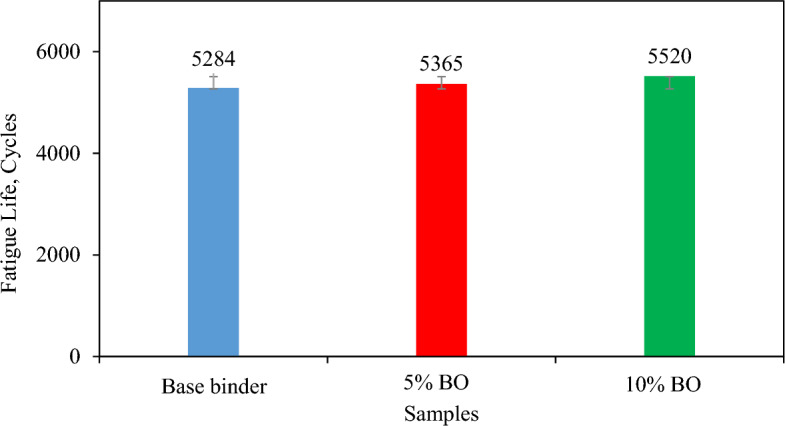
Comparative Analysis of Fatigue Life Cycles for Various Percentages of Bio-Oil Modified Binder.

The progressive weakening of materials due to repetitive loads caused by fatigue cracking leads to the development of cracks and eventual pavement failure^63^. This section presents a discussion on the results of the Four-Point Beam Fatigue Test (FPBFT) in terms of failure cycles, considering the impact of material failure on the formation of fatigue cracks. Figure 17 shows the results that were obtained in stress control mode. In general, the failure cycles increase with the addition of CS bio-oil modified binder to the virgin mixture due to the softening-gain mechanism. When compared to the virgin mixture, the maximum increase is noted for 10% CS bio-oil modified binder concentration in the mixture. The percentage increase of 4.47% and 1.53% is noted at 10% and 5% contents of CS bio-oil modified binder in the virgin mixture, respectively. This rise in fatigue cycles is the function of decreased flexural stiffness as well as initial dissipated energy^22^. The statistical analysis (Table 12) conducted on the loading cycles has shown a significant enhancement in the fatigue resistance of the asphalt binder upon the incorporation of CS bio-oil modified binder (*P*-value < 0.05, null hypothesis rejected).

**Table 12:** Statistical analysis of loading cycles.

Source	df	F	Sig	Significant effect
Corrected Model	2	47.934	< 0.001	
Intercept	1	290,485.068	< 0.001	
Bio-oil modified binder percentage	2	47.934	< 0.001	Yes

## Conclusions

This research examines the impact of various asphalt binder modifications on fatigue, rutting, and moisture damage. The research delves into detailed analyses across different sections, offering thorough interpretation and discussion of the results. The conclusions drawn from these analyses are summarized as follows:

- Asphalt binders modified with CS bio-oil showed significant softening effects, as evidenced by increased penetration values, and decreased softening points. Furthermore, these modified binders were more ductile than virgin binders.
- The DSR test result indicated that the 10% CS bio-oil modified binder performed well against rutting without compromising rutting resistance.
- The CS Bio-oil modified binder binders maintained equivalent PG ratings compared to virgin binder binders, indicating enhanced resilience against fatigue cracking.
- The inclusion of CS bio-oil modified binder weakened moisture resistance, resulting in deterioration of the bond between aggregates and binder, as measured using the BBS and RBT.
- Evaluation of fatigue performance using Dynamic Modulus showed that CS bio-oil modified binder improved fatigue performance similarly to DSR. This enhancement occurred without negatively impacting rutting resistance.
- The WTT and FPBFT tests further supported the conclusions drawn from the analysis of dynamic modulus (i.e., negligible change in rutting resistance and greater improvement in fatigue performance, respectively).

## Data Availability

All data used in this research appear in the submitted article.
