# Author Correction: Bio-oil modified binder derived from cotton stalks as an eco-friendly alternative binder for flexible pavements

**DOI:** 10.1038/s41598-024-74547-6

**Published:** 2024-10-04

**Authors:** Sahib Ullah, Syed Bilal Ahmed Zaidi, Diyar Khan, Ayyaz Fareed, Mohd Rosli Mohd Hasan, Abdalrhman Milad, Basit Ali

**Affiliations:** 1 Department of Civil Engineering, University of Engineering and Technology, Taxila, Pakistan; 2 Doctoral School, Silesian University of Technology, Akademicka 2a, 44-100 Gliwice, Poland; 3 Sustainable Asphalt Research Group (SARG), School of Civil Engineering, Universiti Sains Malaysia (Engineering Campus), 14300 Nibong Tebal, Penang Malaysia; 4 Department of Civil and Environmental Engineering, College of Engineering and Architecture, University of Nizwa, 616 Birkat-al-Mouz, Nizwa, Oman; 5 School of Highway Engineering, Chang’an University, Xi’an, PR China

Correction to: *Scientific Reports*10.1038/s41598-024-62652-5, published online 12 June 2024

The original version of this Article contained an error in the Acknowledgements section.

“This publication is supported under the excellence initiative—research university program under the grant no. 32/014/SDU/10-27-10.1038/s41598-024-62652-509, Silesian University of Technology, Gliwice, Poland and TRC research project BFP/RGP/EI/23/073 University of Nizwa, Oman.”

now reads:

“This publication is supported under the excellence initiative—research university program under the grant no. 32/014/SDU/10-27-09, Silesian University of Technology, Gliwice, Poland and TRC research project BFP/RGP/EI/23/073 University of Nizwa, Oman.”

The original Article has been corrected.

